# Analysis of adverse events and quality of life data for an economic evaluation of adjuvant chemotherapy in colorectal cancer: when can we stop collecting?

**DOI:** 10.1186/1745-6215-12-S1-A41

**Published:** 2011-12-13

**Authors:** Kathleen A Boyd, Andrew H Briggs, Jim Paul, Tim Iveson, Rachel Midgely, Andrea Harkin, Gaynor Bates, Laura Alexander, Jim Cassidy

**Affiliations:** 1 Health Economics & Health Technology Assessment, University of Glasgow, Glasgow, G12 8RZ, UK; 2 Cancer Research UK Clinical Trials Unit Glasgow, Glasgow, G12 0YN, UK; 3 Southampton University Hospitals NHS Trust, Southampton, SO16 6YD, UK; 4 Department of Clinical Pharmacology , Oxford, OX3 7DQ, UK; 5 Oxford Clinical Trials Office, Oxford, OX3 7DQ, UK

## Background

The SCOT colorectal cancer trial is a phase III randomised controlled, multi-centre, non-inferiority trial comparing the efficacy of 12 weeks of chemotherapy versus 24 weeks and the associated toxicity, along with a lifetime economic analysis. The economic analysis will use prospective trial data to inform a lifetime cost-effectiveness model.

Quality of life and toxicity data are only collected in a proportion of patients as the sample size required for these comparisons is much smaller than that required to show a significant difference in efficacy. This paper reports on an analysis of adverse events (AEs) and quality of life data, undertaken to determine whether it was appropriate to stop collecting quality of life data from new recruits. Do we have a large enough sample to detect whether the AEs and severity of AEs impact on quality of life?

## Methods

Statistical analysis of data from the SCOT trial of patients with fully resected stage III colorectal cancer or fully resected high-risk stage II disease. Data collection commenced June 2008. EQ-5D and AE data was recorded at baseline, each treatment cycle and at follow-up intervals until December 2010. This interim dataset was analysed in STATA, using univariate and multivariate regression.

## Results

Univariate analysis showed that the majority of AEs significantly reduce quality of life (significant at 3% or less); however, multivariate analysis was inconclusive as AEs were highly correlated with one another. Uni- and multivariate regressions were re-run using indicator variables for AE grade groups for each specific adverse event type, and showed a highly significant (1% level) negative impact on quality of life as severity of grade increased, particularly for the adverse events Diarrhoea (-0.04 grade 1/2, -0.09 grade 3/4), Fatigue (-0.02 grade 1/2), Nausea (-0.05 grade 1/2, -0.14 grade 3/4), Neuropathy sensory (-0.02 grade 1/2, -0.19 grade 3/4), and Vomiting (-0.05 grade 1/2). Quality of life had a significant negative relationship with severity of AE grade, regardless of the specific event (0.017 grade 1, -0.058 grade 2, -0.062 grade 3, -0.145 grade 4).

## Conclusions

Adverse events impact negatively on quality of life; however, multivariate regression was inconclusive due to multicolinearity between the adverse events. There was strong evidence to show that the AEs and AE grades significantly reduced quality of life using univariate analysis and therefore we can be confident that the sample from June 2008-Dec 2010 is sufficient and can stop collecting such data from new recruits.

